# Reference intervals for thyroid hormones for the elderly population and their influence on the diagnosis of subclinical hypothyroidism

**DOI:** 10.5937/jomb0-39570

**Published:** 2023-03-15

**Authors:** Jingxuan Fu, Yidan Wang, Yunyi Liu, Qinfei Song, Jingrong Cao, Wang Peichang

**Affiliations:** 1 Capital Medical University, National Clinical Research Center for Geriatric Diseases, Xuanwu Hospital, Department of Clinical Laboratory, Beijing, China

**Keywords:** subclinical hypothyroidism, elderly, thyroid hormones, reference intervals, subklinički hipotireoza, starije osobe, tiroidni hormoni, referentni intervali

## Abstract

**Background:**

This study aims to establish reference intervals (RIs) for thyroid hormones in the elderly population and analyze their influence on the prevalence of subclinical hypothyroidism.

**Methods:**

Thyroid hormone records of subjects who underwent routine health checkup at our hospital between 2018 and 2020 were analyzed. Thyroid stimulating hormone (TSH), total triiodothyronine, total thyroxine, free triiodothyronine (FT3), and free thyroxine (FT4) levels were compared between young and elderly subjects. Thresholds of these thyroid hormones were established for elderly subjects.

**Results:**

A total of 22,207 subjects were included. Of them, 2,254 (10.15%) were aged ≥ 65 years. Elderly subjects had higher TSH, and lower FT3 and FT4 levels when compared with young subjects. In the elderly group, the RIs for TSH, FT3 and FT4 were 0.55-5.14 mIU/L, 3.68-5.47 pmol/L, and 12.00-19.87 pmol/L, respectively. The age and sex specific RIs for TSH were 0.56-5.07 mIU/L for men and 0.51-5.25 mIU/L for women. With whole-group RIs and age and sex-specific RIs for elderly people, the prevalence of subclinical hypothyroidism was 9.83% and 6.29% (p < 0.001), respectively.

**Conclusions:**

Elderly individuals had higher TSH levels than young individuals. Our study indicated that establishing specific RIs for elderly individuals is needed. This has implications for the diagnosis and management of subclinical hypothyroidism in the elderly population.

## Abstract (sr)

**Uvod:**

Ova studija ima za cilj da utvrdi referentne intervale (RI) za tiroidne hormone u starijoj populaciji i analizira njihov uticaj na prevalenciju subkliničkog hipotireoze.

**Metode:**

Analizirani su podaci o hormonima štitaste žlezde ispitanika koji su bili podvrgnuti rutinskom zdravstvenom pregledu u našoj bolnici u periodu od 2018. do 2020. godine. Tireostimulišući hormon (TSH), ukupni trijodtironin, ukupni tiroksin, slobodni trijodtironin (FT3) i nivo slobodnog tiroksina (FT4) upoređivani su između mladih i starijih ispitanika. Pragovi vrednosti ovih tiroidnih hormona su utvrđeni za starije osobe.

**Rezultati:**

Uključeno je ukupno 22.207 ispitanika. Od njih 2.254 (10,15%) bilo je staro ≥ 65 godina. Stariji subjekti su imali veći TSH i niže nivoe FT3 i FT4 u poređenju sa mladim subjektima. U starijoj grupi, RI za TSH, FT3 i FT4 bili su 0,55-5,14 mIU/L, 3,68-5,47 pmol/L i 12,00-19,87 pmol/L, respektivno. RI za TSH bio je 0,56-5,07 mIU/L za muškarce i 0,51-5,25 mIU/L za žene. Kod RI cele grupe i RI specifičnih za uzrast i pol za starije osobe, prevalencija subkliničkog hipotireoze bila je 9,83% i 6,29% (p < 0,001), respektivno.

**Zaključak:**

Starije osobe su imale veći nivo TSH od mladih osoba. Naša studija je pokazala da je potrebno uspostavljanje specifičnih RI za starije osobe. Ovo ima implikacije na dijagnozu i lečenje subkliničkog hipotireoze kod starije populacije.

## Introduction

According to the Seventh National Population Census of China in 2020, which was released by the National Bureau of Statistics of China, the Chinese population aged ≥ 60 years numbered over 264 million (264,018,766), accounting for 18.70% of the total population, and the population aged ≥ 65 years accounted for 13.50% of the total population [1]. Compared with 2010, the proportion of the population aged ≥ 60 years and ≥ 65 years increased by 5.44% and 4.63%, respectively [1]. China's population is rapidly aging, with a high prevalence of age-related diseases; this poses a significant challenge to the Chinese healthcare system. The morphology of the thyroid gland and its synthetic and secretory functions change physiologically and pathologically with age. Thyroid volume shrinks gradually after 50 years of age, and is associated with the fibrosis, atrophy, and lymphocytic infiltration of the gland [2]. Compared with young people, iodine level in the elderly population is reduced, which may be related to a low-salt diet and decreased absorption capacity caused by comorbidities and drugs [2]. It has been reported that with normal aging, thyroid stimulating hormone (TSH) level increases, free thyroxine (FT4) level remains stable or increases slightly, and total triiodothyronine (TT3) and free triiodothyronine (FT3) levels decrease [3] [4] [5].

As reported previously, the prevalence of hypothyroidism ranges from about 12–18% in the elderly population, comparatively higher than the 3.7–9.5% in the general population [6] [7] [8] [9]. Subclinical hypothyroidism with elevated TSH and normal FT4 levels is largely proportional to the prevalence of hypothyroidism. This relationship is reflected in a study based on the National Health and Nutrition Examination Survey (NHANES), which showed that the prevalence rate of subclinical hypothyroidism increased in older age categories [7]. The age categories of 12–49, 50–79, and > 80 years showed prevalence rates of 2.2%, 5.0%, and 10.9%, respectively [7]. The prevalence rate in NHANES is based on reference intervals (RIs) for the general population. The clinical manifestations of hypothyroidism in the elderly are often diverse, lack specificity, and are also affected by comorbidities [10]. Therefore, the diagnosis of hypothyroidism and subclinical hypothyroidism in the elderly is dependent mainly on serum thyroid function tests, including TSH, FT4, and FT3.

TSH level increases gradually with age and can be considered a part of the normal human aging process [8]. Nevertheless, most hospitals still use a unified reference interval for people of different age categories, established based on the general population, which may increase cases of misdiagnosed hypothyroidism, especially subclinical hypothyroidism, in elderly people. Consequently, unnecessary thyroxine replacement therapy is prescribed, which has no benefits and increases the risk of developing atrial fibrillation and fractures [11] [12].

In this study, we analyzed the influence of age on thyroid hormone levels and established RIs for thyroid function tests in the Chinese elderly population. Further, we also analyzed the influence of these RIs on the diagnosis of hypothyroidism.

## Materials and methods

### Data collection

Thyroid hormone records of patients who underwent routine health checkup at Xuanwu Hospital, Capital Medical University, Beijing, China, between January 2018 and July 2020 were extracted from the laboratory information system. The extracted data included sex, age of routine health checkups, and the TSH, TT3, TT4, FT3, and FT4 values. We extracted 24,780 subjects from the laboratory information system. The exclusion criteria were as follows: subjects with missing information (including TSH, TT3, TT4, FT3, FT4, sex, and age), TSH out of detection range (>150 mIU/L and <0.008 mIU/L), <18 years old, pregnant females, and abnormal anti-thyroid peroxidase antibody (>60,000 IU/L) or anti-thyroglobulin antibody (>41,000 IU/L) [13]. Finally, 24,609 subjects were included in the study.

### Laboratory measurements and quality assurance

Fasting blood samples were drawn into red-capped vacuette blood collection tubes (Greiner Bio-One, Kremsmünster, Austria) and we used it throughout the study. All samples were centrifuged at 3000 rpm for 10 min. Sample collection, transportation, and pretreatment were performed strictly according to the standard operating procedures of our laboratory based on the National Guide to Clinical Laboratory Procedures and The Clinical & Laboratory Standards Institute. Fasting blood samples were collected from 7:30 am to 10:30 am. Centrifugation was completed within 2 hours of collection. Samples with lipemia and hemolysis were excluded based on the unqualified sample process. All samples were tested using a Siemens ADVIA Centaur XP Immunoassay System (Siemens Healthcare Diagnostics Inc., Tarrytown, NY, USA), and regular calibration was conducted using the matching reagent as suggested by the manufacturer. To ensure the measurement quality, internal quality control was performed using quality control materials (BIO RAD lyphochek Immunoassay Plus Control) before sample data were collected every day. We also participated in external quality assessments by the National Center for Clinical Laboratories to ensure precision.

### Statistical Analysis

We used the Tukey method to remove outliers [14]. The median, first quartile (Q1), third quartile (Q3), and interquartile range (IQR) were calculated. Datapoints below Q1–1.5*IQR or above Q3+1.5*IQR were considered outliers. The Kolmogorov–Smirnov test was used to measure data distribution. Normally distributed data are presented as the mean ± SD. Non-normally distributed data are described as the median and IQR. For non-normally distributed data, the Mann–Whitney U test and Kruskal–Wallis test were used to compare differences between groups, as appropriate. The thresholds for TSH, TT3, TT4, FT3, and FT4 were established as the 2.5th and 97.5th percentiles of the distribution for the reference subjects. We analyzed the prevalence of hypothyroidism with whole-group RIs, and our recommended age- and sex-specific RIs for elderly people. Overt hypothyroidism was defined as high TSH and low TT4 or FT4 levels. Subclinical hypothyroidism was defined as high TSH and normal TT4 and FT4 levels. Elderly individuals were defined as those aged ≥ 65 years. When analyzing the prevalence of hypothyroidism, all subjects aged ≥ 65 years in our database were considered. SPSS 23 software (IBM Inc., Armonk, NY, USA) was used to analyze the data. Results with p < 0.05 were considered significant.

## Results

The data of 24,780 subjects were extracted from the laboratory information system. After removing data that fell under the exclusion criteria (n=171), 24,609 subjects were included in the study. Then, we excluded 2402 outliers using the Tukey method; 22,207 subjects were finally included. Of them, 2,254 (10.15%) were aged ≥ 65 years. The Kolmogorov-Smirnov test showed that TSH, TT3, TT4, FT3 and FT4 levels were non-normally distributed. The median (IQR) TSH, TT3, TT4, FT3, FT4 levels were 1.91 (1.38–2.65) mIU/L, 1.60 (1.45-1.77) nmol/L, 98.04 (86.43–108.36) nmol/L, 4.79 (4.45–5.14) pmol/L and 15.74 (14.45–17.03) pmol/L, respectively. The basic characteristics of the subjects are listed in Table 1.

**Table 1:** The basic characteristics of subjects and distribution among different gender and age groups TSH, TT3, TT4, FT3, and FT4 are presented as median.<br>Abbreviations: TSH, thyroid stimulating hormone; TT3, total triiodothyronine; TT4, total thyroxine; FT3, free triiodothyronine; FT4, free thyroxine.

Characteristics	Number	TSH<br>(mIU/L)	p	TT3<br>(nmol/L)	p	TT4<br>(nmol/L)	p	FT3<br>pmol/L)	p	FT4<br>(pmol/L)	p
Gender											
Men	10548	1.80	<0.001	1.65	<0.001	96.75	0.001	5.04	<0.001	16.38	<0.001
Women	11659	2.02		1.57		98.04		4.57		15.22	
Age											
18–29	2781	1.94	<0.001	1.60	<0.001	98.04	0.001	4.90	<0.001	16.25	<0.001
30–39	6545	1.87		1.59		95.46		4.87		15.87	
40–49	5206	1.89		1.59		96.75		4.74		15.61	
50–64	5421	1.97		1.62		99.33		4.76		15.61	
>=65	2254	1.96		1.60		100.62		4.57		15.48	
Young/Elderly											
Young (18–64)	2254	1.91	0.002	1.60	0.862	96.75	<0.001	4.80	<0.001	15.74	<0.001
Elderly (>=65)	19953	1.96		1.60		100.62		4.57		15.48	

### Distribution of thyroid hormones according to age

**Figure 1:**
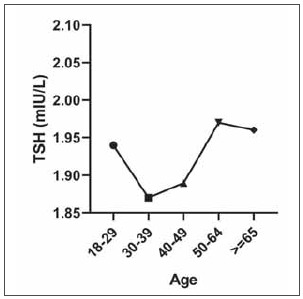
The distribution of thyroid stimulating hormone (TSH) with age. Plots showing the median TSH for each of the changes according to different age group.

As shown in Table 1, FT3 and FT4 levels declined with age. TSH (Figure 1) and TT4 levels tended to increase with age after 30–39 years. TT3 showed no apparent trend with age.

**Figure 2:**
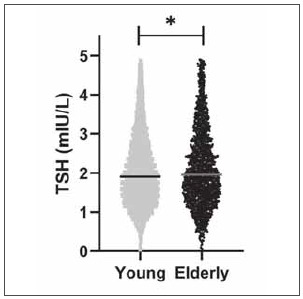
Thyroid stimulating hormone (TSH) distribution in young and elderly individuals. Plots showing the median TSH of young individuals (aged 18–64 years) and elderly individuals (aged ≥ 65 years). *p<0.05.

As shown in Table 1 and Figure 2, elderly subjects had higher TSH (median 1.96 vs. 1.91 mIU/L), and TT4 (median 100.62 vs. 96.75 nmol/L) levels, lower FT3 (median 4.57 vs. 4.80 pmol/L) and FT4 (median 15.48 vs. 15.74 pmol/L) levels when compared with those of younger subjects. TT3 (median 1.60 vs. 1.60 nmol/L) levels were similar between the two groups.

### RIs in elderly subjects

**Figure 3:**
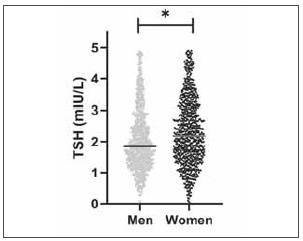
Gender distribution of thyroid stimulating hormone (TSH) in elderly individuals. Plots showing the median TSH of men and women in elderly individuals. *p<0.05.

As shown in Table 2, the RIs for TSH, TT3, TT4, FT3, and FT4 were 0.64–4.31 mIU/L, 1.17–2.13 nmol/L, 67.08–131.58 nmol/L, 3.85–5.85 pmol/L, and 12.38–19.74 pmol/L in whole group, respectively. In the elderly group, the RIs for TSH, FT3 and FT4 were 0.55–5.14 mIU/L, 3.68–5.47 pmol/L, and 12.00–19.87 pmol/L, respectively. In the elderly group, women had higher TSH (median, 2.18 vs. 1.85 mIU/L, p<0.001, Figure 3) and TT4 (median, 104.49 vs. 98.04 nmol/L, p<0.001) levels and lower FT3 (median, 4.47 vs. 4.67 pmol/L, p<0.001) and FT4 (median, 15.22 vs. 15.74 pmol/L, p<0.001) levels. TT3 levels were similar between women and men (median, 1.60 vs. 1.60 nmol/L, p=0.872).

**Table 2:** Whole-group RIs and age- and sex-specific RIs for elderly people for thyroid hormones Whole-group RIs and age- and sex-specific RIs for elderly people were established as the P2.5 and P97.5.<br>Abbreviations: TSH, thyroid stimulating hormone; TT3, total triiodothyronine; TT4, total thyroxine; FT3, free triiodothyronine; FT4, free thyroxine; RIs, reference intervals.

		TSH (mIU/L)	TT3 (nmol/L)	TT4 (nmol/L)	FT3 (pmol/L)	FT4 (pmol/L)
Whole-group RIs		0.64–4.31	1.17–2.13	67.08–131.58	3.85–5.85	12.38–19.74
Age- and<br>sex-specific RIs<br>for elderly people	Men	0.56–5.07	1.08–2.14	61.92–135.45	3.73–5.53	12.13–20.00
	Women	0.51–5.25	1.17–2.13	72.24–139.32	3.65–5.31	11.74–19.22

After excluding 278 outliers, 2377 subjects were used to establish RIs for elderly individuals. The RIs for elderly women and men are shown in Table 2. The age- and sex-specific RIs for TSH were 0.56–5.07 mIU/L for men and 0.51–5.25 mIU/L for women.

A total of 2,655 elderly subjects were included to analyze the prevalence of overt and subclinical hypothyroidism. Applying whole-group RIs and age- and sex-specific RIs for elderly people, 2218 and 2375 (p<0.001) subjects had normal TSH, respectively. With age-and sex-specific RIs, more subjects had normal TSH. As shown in Table 3, with whole-group RIs and age- and sex-specific RIs for elderly people, the prevalence of hypothyroidism (including overt or subclinical hypothyroidism) was 12.09% and 7.83% (p< 0.001), respectively; prevalence of subclinical hypothyroidism was 9.83% and 6.29% (p< 0.001), respectively; and the prevalence of overt hypothyroidism was 2.30% and 1.54% (p= 0.056), respectively. Less individuals were diagnosed with subclinical hypothyroidism with age- and sex-specific RIs for elderly people.

**Table 3:** Prevalence of overt and subclinical hypothyroidism in elderly individuals with whole-group RIs and age- and sex-specific RIs for elderly people

	Overt<br>hypothyroidism	p	Subclinical<br>hypothyroidism	p	Overt or subclinical<br>hypothyroidism	p
Whole-group RIs	60 (2.30%)	0.056	261 (9.83%)	< 0.001	321 (12.09%)	<0.001
Age- and sex-specific RIs<br>for elderly people	41 (1.54%)		167 (6.29%)		208 (7.83%)	

## Discussion

In the present study, we found that elderly people had higher TSH levels than young people; this is similar to previous reports [3] [4] [5] [14]. NHANES III, a cross-sectional study that included 16,533 individuals, demonstrated that TSH concentration increases with age. For individuals aged 20–29 and > 80 years, the median TSH level was 1.28 mIU/L and 2.08 mIU/L, respectively [15]. In a longitudinal study of a community-based cohort, TSH, FT4, thyroglobulin antibodies, and thyroid peroxidase were measured in 1,100 participants in 1981 and 1994 [4]. After excluding participants with thyroid disease or thyroid antibodies, 908 participants were analyzed. The mean baseline age (in 1981) was 45 years. During 13 years of follow-up, TSH increased from 1.49 to 1.81 mIU/L [4]. Another study included 843 participants > 85 years from the Cardiovascular Health Study All Stars Study; TSH, FT4, TT3, and thyroid peroxidase antibodies were measured in 1992–1993 and 2005–2006. During follow-up, there was a statistically significant 13% increase in TSH levels [3].

It has been reported that FT4 remains stable or increase slightly with age; TT3 and FT3 levels decrease with age [3] [4] [14]. In the Cardiovascular Health Study All Stars Study, there was a 1.7% increase in FT4 and a 13% decrease in TT3 over 13 years [3]. In a community-based cohort longitudinal study, the mean FT4 remained unchanged (16.6 pmol/L vs. 16.6 pmol/L) at the 13-year follow-up [4]. Similar to these studies, we found that FT3 levels decreased with age. However, in the present study, elderly subjects had lower FT4 levels than young subjects, and TT3 was similar between the two groups, which is different from previous reports. There are several reasons for this. First, the research methods used were different. The Cardiovascular Health Study All Stars Study and the study by Waring et al. [3] and Bremner et al. [4] were cohort studies. The present study was a cross-sectional study. Second, the study population differed. Previous studies were community-based; our study was based on data acquired from routine health checkups [3] [4].

In the present study, we devised RIs for thyroid hormone levels in the elderly. We recommended 0.56–5.07 mIU/L for men and 0.51–5.25 mIU/L for women as RIs for TSH in the elderly. In previous studies, RIs recommended for the elderly were 0.32–5.55 mIU/L; 0.53–5.24 mIU/L (men), and 0.34–5.73 mIU/L (women); 0.28–6.53 mIU/L (men) and 0.44–6.8 mIU/L (women); and 0.58–6.2 mIU/L (men) and 0.54–6.07 mIU/L (women) [16] [17] [18] [19]. The recommended RIs for elderly subjects were different in these studies. There are several reasons for this. First, race, seasons, temperature, sampling time, and iodine intake influence thyroid hormone levels [8] [20]. Second, the equipment and reagents influenced thyroid hormone testing. Third, in these studies, the definition of elderly differed, including >65, >70, and >80 years [16] [17] [18] [19]. As many factors influence RIs, it is meaningful to establish new RIs, although several RIs for elderly subjects have been reported, as shown above. The newly established RIs are more applicable in our region and institutes using the same automatic method. In the 2019 French Endocrine Society consensus statement, for individuals > 60 years, the upper limit of RIs is the patient's age (decade) divided by 10: for example, TSH ≤ 7 for 70-year-old, or ≤ 8 mIU/L for 80-year-old individuals [21].

Symptoms of subclinical hypothyroidism are non-specific or atypical in the elderly; therefore, the diagnosis of subclinical hypothyroidism is dependent on thyroid hormone levels. The prevalence of subclinical hypothyroidism depends on the upper limit of TSH levels. The upper limits of RIs for TSH were 5.07 mIU/L for men and 5.25 mIU/L for women in the elderly. This is higher than 4.31 mIU/L, which is whole-group upper limit for the general population. With a higher TSH upper limit reference interval, fewer elderly individuals will be diagnosed with subclinical hypothyroidism. After applying whole-group RIs and age- and sex-specific RIs for elderly people, the prevalence rates of subclinical hypothyroidism were 9.83% and 6.29% (p< 0.001), respectively; the prevalence of subclinical hypothyroidism was reduced by approximately 3.5%. Considering that there are more than 190 million elderly individuals in China, the change in RIs for TSH will prevent many such patients from being misdiagnosed with subclinical hypothyroidism. It has been reported that in elderly individuals with TSH <7 mIU/L, the cardiovascular, musculoskeletal, and neurocognitive risks did not increase [21] [22]. Moreover, mortality in elderly individuals with subclinical hypothyroidism is not increased [3]. In the 2019 French Endocrine Society consensus statement, levothyroxine replacement therapy is not recommended for patients with TSH < 10 mIU/L [21]. A higher TSH upper limit of normal will result in fewer elderly individuals being diagnosed with subclinical hypothyroidism and reduce unnecessary replacement therapy.

This study had some limitations. Firstly, when we establish RIs for thyroid hormones, we need to exclude individuals with goiter and known thyroid disease. In the present study, these individuals were not completely excluded. Instead, outliers were excluded with the Tukey method. The RIs established in the present study cannot be directly used. Before clinical application, we need to establish age- and sex-specific RIs with strict inclusion and exclusion criteria. Secondly, we used the same population to establish RIs and calculate the prevalence hypothyroidism. Calculation of the prevalence of hypothyroidism with an independent population is needed.

## Conclusion

Elderly individuals had higher TSH levels than young individuals. Our study indicated that establishing specific RIs for elderly individuals is needed. This has implications for the diagnosis and management of subclinical hypothyroidism in the elderly population.

## Dodatak

### Ethics approval and informed consent

All procedures performed in studies involving human participants were in accordance with the ethical standards of the institutional and/or national research committee and with the 1964 Helsinki declaration. The study was approved by the Bioethics Committee of Xuanwu Hospital, Capital Medical University. Written informed consent from the study subjects was not acquired because: this is a retrospective study, the research could not practicably be carried out without the requested waiver or alteration. The research involves no more than minimal risk to the subjects; the waiver or alteration will not adversely affect the rights and welfare of the subjects. We confirm that patient data confidentiality is protected strictly. There is no identifiable information in the paper.

### Funding

This study was supported by the National Nature Science Foundation of China (grant number 81901406), Beijing Municipal Administration of Hospitals’ Ascent Plan (DFL20180803), and Beijing High-level Public Health Technical Talent Development Plan (2022-01-023).

### Abbreviations

TSH, thyroid stimulating hormone;<br>TT3, total triiodothyronine;<br>TT4, total thyroxine;<br>FT3, free triiodothyronine;<br>FT4, free thyroxine;<br>RIs, reference intervals;<br>Q1, first quartile;<br>Q3 third quartile;<br>IQR, interquartile range

### Conflict of interest statement

All the authors declare that they have no conflict of interest in this work.
